# The crystal cargo provides a chronicle of pre-caldera dynamics in mafic volcanic systems: insights from Colli Albani

**DOI:** 10.1007/s00445-025-01865-6

**Published:** 2025-08-30

**Authors:** Mónica Ágreda-López, Alessandro Musu, Corin Jorgenson, Martin Šala, Guido Giordano, Luca Caricchi, Ciprian Stremtan, Maurizio Petrelli

**Affiliations:** 1 Dept. of Physics and Geology, University of Perugia, Piazza Università, 1, Perugia, 06123 Italy; 2 Dept. of Lithospheric Research, University of Vienna, Josef-Holaubek-Platz 2, Vienna, 1090 Austria; 3 Dept. of Earth Sciences, University of Geneva, Rue de Maraîchers, 13, Geneva, 1205 Switzerland; 4 Dept. of Civil & Environmental Engineering, University of Strathclyde, James Weir Building Level 5, 75 Montrose Street, Glasgow, G11XJ Scotland; 5 Dept. of Analytical Chemistry, National Institute of Chemistry, Hajdrihova 19, Ljubljana, 1000 Slovenia; 6 Dept. of Science, Geology, Università Degli Studi Roma Tre, S. Murialdo 1, Rome, 00146 Italy; 7 Teledyne Photon Machines, 384 Gallatin Park Drive, Bozeman, MT 59715 USA

**Keywords:** Mafic-alkaline magmatism, Caldera-forming eruption, Clinopyroxene, Machine learning

## Abstract

Understanding the processes leading up to caldera-forming eruptions is essential for identifying precursory signals of catastrophic events. While these phenomena have been extensively studied in silicic systems, mafic volcanoes present unique challenges. Indeed, the high eruptive temperatures of mafic magmas might imply short storage in the cold upper crust and, thus, short periods of unrest preceding eruption, which could challenge our capacity to mitigate the impact of an imminent event. In this study, we present new textural data, major- and trace-element analyses, and quantitative trace-element maps of the crystal cargo from an effusive to mildly explosive sequence (the Fontana Centogocce Formation) and the subsequent caldera-forming phase (the Villa Senni Formation) at the Colli Albani volcano in Italy. By integrating well-established and data-driven approaches, we constrain the processes and dynamics that drive the transition from mildly explosive to highly explosive activity in the studied magmatic sequences. Our findings reveal that the effusive to mildly explosive eruptions preceding the caldera-forming event were fed by multiple magma reservoirs emplaced at shallow crustal levels ($\sim$1–4 kbar). Following a quiescent period recorded by a paleosol, more primitive magma rose directly from the mantle and accumulated at multiple crustal levels. The ascent of one of these magma pulses ultimately triggered Colli Albani’s last caldera-forming eruption.

## Introduction

Understanding how caldera-forming eruptions work and how eruptive styles change over time is a complex, challenging, and highly debated topic in petrology and volcanology (Roggensack et al. 1997; Lyons et al. 2010; Andújar and Scaillet 2012; Tuffen et al. 2013; Stoppa et al. 2017; Cassidy et al. 2018; Forni et al. 2018; Schipper et al. 2019; Bouvet de Maisonneuve et al. 2021). It requires capturing the essence of several processes and dynamics that occur across diverse spatial and temporal scales. Some of these processes are modulated by intrinsic properties governing magma rheology, such as magma composition, volatile content, oxygen fugacity, and magma storage conditions (Iacovino et al. 2013; Cross et al. 2014; Newcombe et al. 2020; Allison et al. 2021). Additional factors controlling eruptive dynamics include the frequency, rate and volume of episodically injected primitive magmas, volatile exsolution at shallow crustal levels, the assimilation of host silicate or carbonate rocks, magma mixing, fluid interactions, or the segregation and subsequent migration of melt pockets from deep to shallow reservoirs, along with conduit dynamics (Iacono-Marziano et al. 2007; Gaeta et al. 2009; Avanzinelli et al. 2009; Boari et al. 2009; Freda et al. 2011; Di Rocco et al. 2012; Blythe et al. 2015; Caricchi et al. 2021; Giordano and Caricchi 2022).

Much effort has been dedicated to understanding large caldera-forming eruptions in silicic systems, particularly in addressing key questions such as: What are the precursors of a caldera-forming eruption? (Kennedy et al. 2018; Keller et al. 2023) How does the magma plumbing system behave before a caldera-forming eruption? (Jellinek and DePaolo 2003; Bachmann and Bergantz 2008) Which processes influence the transition between low- and high-energy eruptive phases? (Woods and Koyaguchi 1994; Cassidy et al. 2018; Forni et al. 2024) How can signs of unrest be interpreted? (Geshi et al. 2023; Keller et al. 2023). For example, Bouvet de Maisonneuve et al. (2021) summarized the evolution of silicic caldera-forming systems through a series of recursive processes (i.e., a “caldera cycle”). According to Bouvet de Maisonneuve et al. (2021), a caldera cycle includes (1) a typically long build-up phase of mushy systems in the upper crust (i.e., incubation and maturation); (2) a high-energy phase (i.e., the catastrophic caldera-forming eruption); and (3) a series of post-collapse events (i.e., the recovery phase).

On the other hand, a comprehensive understanding of the processes leading to caldera-forming eruptions in mafic systems is still lacking. Mafic volcanoes are usually perceived as much less dangerous than their silicic counterparts since these magmas commonly erupt effusively because of their low viscosity. However, mafic magmas can sometimes shift from effusive to explosive activity with little to no warning (e.g., Andronico et al. 2009). More dangerously, this shift can generate large-volume caldera-forming eruptions, as is the case at Masaya (Nicaragua; Walker et al. 1993; Pérez et al. 2009; Bamber et al. 2022), Taal (Philippines; Delos Reyes et al. 2018), Llaima (Chile; Marshall et al. 2022; Bernard et al. 2022), and Colli Albani (Italy; Giordano et al. 2010; Jorgenson et al. 2024) volcanoes. There is evidence that, in mafic systems, the build-up phase preceding a catastrophic eruption should be significantly shorter than in silicic systems, as the higher eruptive temperatures of mafic magmas imply shorter residence times within the colder upper crust (Annen and Sparks 2002; Sparks et al. 2019; Costa et al. 2020; Petrelli and Zellmer 2020; Jorgenson et al. 2024). Therefore, understanding the processes and dynamics occurring in such short periods of unrest preceding a catastrophic eruption, although challenging, is crucial to mitigate the impact of future events.

Recent studies have demonstrated that accurate petrologic investigations on the crystal cargo of volcanic eruptions can help to elucidate pre-eruptive dynamics and provide insights into the architecture of volcanic plumbing systems (Astbury et al. 2018; Ubide and Kamber 2018; Nicotra et al. 2020; Corsaro and Miraglia 2022; Li et al. 2024; D’Mello et al. 2024; Zellmer et al. 2024). Here, we investigate clinopyroxene (Cpx) crystals from different eruptions belonging to the Colli Albani volcano (Italy). We selected this volcanic system since it represents a perfect example of a mafic system, able to shift from effusive to caldera-forming eruptions (Giordano et al. 2010; Jorgenson et al. 2024). Indeed, its eruptive record shows several cycles of effusive to explosive transitions, producing both mildly explosive eruptions and large-volume ignimbrites (>10 km$^3$ DRE; dense-rock equivalent outflow; Giordano et al. 2010), making it an ideal case study to evaluate the processes governing the transition between effusive and caldera-forming mafic eruptions (Giordano et al. 2010). In this contribution, we focus on an effusive to mildly explosive unit, i.e., Fontana Centogocce (SLV) Formation and the subsequent caldera-forming eruptive event of Villa Senni (VSN; $\sim$355 ka; Karner et al. 2001). We present major and trace element data, as well as quantitative trace element maps on both SLV and VSN. We integrate classic petrography, mineral chemistry, and machine learning approaches to combine multiple sources of information. Using cluster analysis, we blend textural observations, microanalytical measurements of major and trace elements, and high-resolution elemental maps to achieve a detailed reconstruction of the volcanic feeding system architecture and pre-eruptive dynamics. Our main aim is to shed new light on the processes governing the transition from effusive and mildly explosive eruptions to caldera-forming events in mafic systems.

## Geological setting and study area

Colli Albani is an ultrapotassic caldera complex located $\sim$30 km SE of Rome (Giordano et al. 2010). This volcano is part of the Roman Magmatic Province, which is an NW-SE extensional chain of ultrapotassic volcanic districts developed during the Pleistocene along the Tyrrhenian margin of Italy (Marra et al. 2004). Colli Albani displays a range of eruptive styles from caldera-forming to Plinian explosive paroxysms and mildly explosive-effusive eruptions (Giordano et al. 2010). All Colli Albani volcanic products are mafic-ultrapotassic rocks, mostly K-foiditic and plagioclase-free (Gaeta et al. 2006). The composition of the magmas from Colli Albani ranges from melilite-bearing leucitites to tephrites and tephri-phonolites (Boari et al. 2009; Conticelli et al. 2010). The main mineral phases are leucites and clinopyroxenes with a range of composition from diopside to Ca Tschermak’s and Ca-Fe Tschermak’s-enriched diopside (Gaeta et al. 2006).

Colli Albani has been active since about 600 ka (Karner et al. 2001). The volcanic activity history has been divided into three main periods: the “Vulcano Laziale” period, the “Tuscolano-Artemisio-Faete” period, and the “Via dei Laghi” period. In the Vulcano Laziale period ($\sim$600 ka to $\sim$355 ka), volcanism was highly explosive and produced seven intermediate to large-volume ignimbrites (Giordano et al. 2010; Vinkler et al. 2012), emplaced with average intervals of $\sim$40 ka (Giordano et al. 2010). Following each ignimbrite eruption, the volcanic activity was mainly effusive to mildly explosive, concentrated along peri-caldera fissure systems.

The last major caldera-forming event took place at $\sim$355 ka, during the Villa Senni ignimbrites emplacement (18 km$^3$ DRE outflow and 10 km$^3$ DRE intracaldera; Giordano et al. 2010). The Villa Senni (VSN) Formation is divided into a basal fallout (VSN0) and two main ignimbrites: Tufo Lionato (VSN1) and Pozzolanelle (VSN2), with ages of 355 ± 2 ka and 357 ± 2 ka, respectively (Freda et al. 1997; Karner et al. 2001). Before the VSN formation, effusive to mildly explosive events occurred, recorded by the Fontana Centogocce (SLV) Formation. SLV consists of fall deposits, lava flows, and pyroclastic deposits that are located stratigraphically between the Pozzolane Nere (407 ± 4 ka) and the Villa Senni ignimbrites (355–357 ± 2 ka) (Karner et al. 2001; Giordano et al. 2010).

**Fig. 1:**
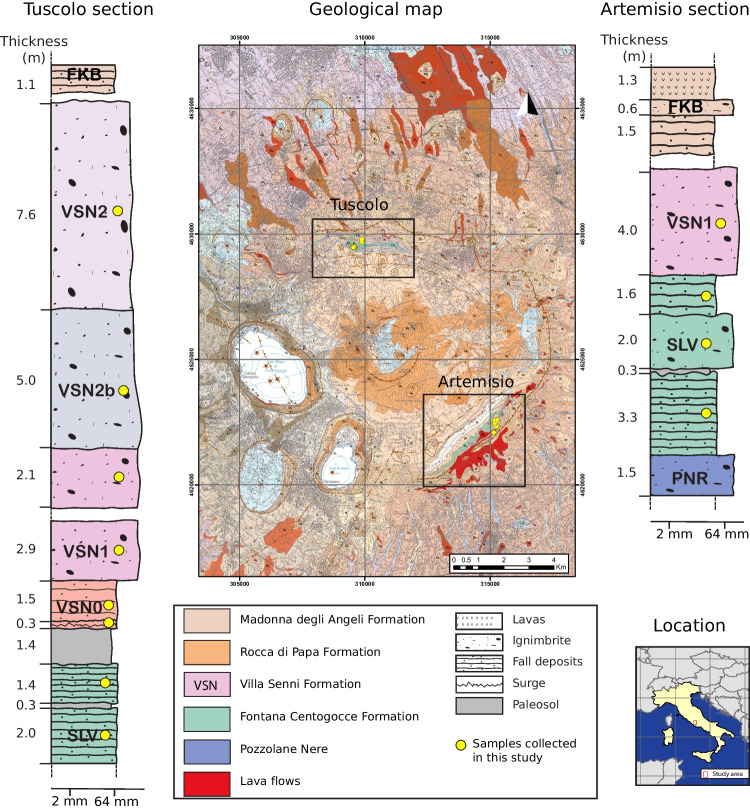
*Map and location of the studied area*. Geological map of Colli Albani volcano modified from Giordano et al. (2010) and stratigraphic columns from Tuscolo and Artemisio sections investigated in this study. Yellow points reflect the exact location where samples were collected on the map and approximate sampling locations along each stratigraphic unit. Sampling was conducted at the bottom, middle, and top of each unit where possible

**Fig. 2:**
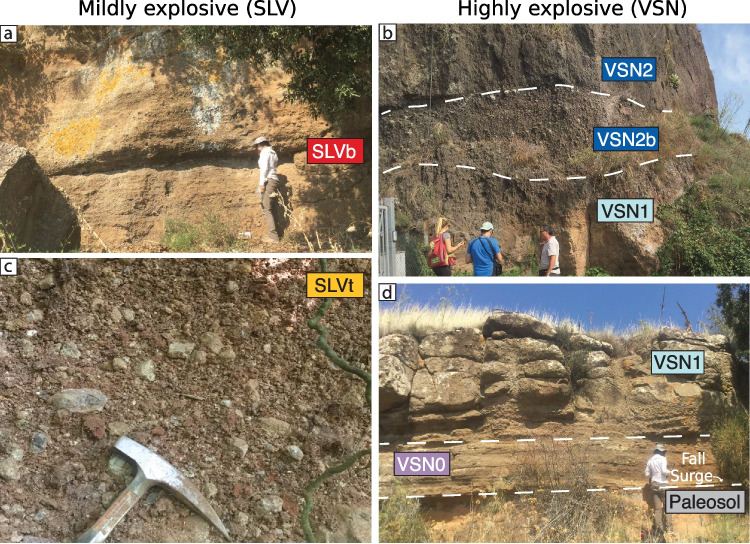
*Field photographs*. **a** and **c** SLV in “Tuscolo” and “Artemisio” localities, respectively. **b** and **d** VSN in “via di Fioranello” and “Tuscolo” localities, respectively. VSN, Villa senni Formation; SLV, Fontana Centogocce Formation

Following the eruption of VSN, the volcanic activity at Colli Albani shifted to smaller volume, effusive to mildly explosive eruptions only. The most recent eruption at Colli Albani occurred $\sim$23 ka at the Albano maar, followed by phreatic activity throughout the Holocene and historical times (Giordano et al. 2010; Funiciello et al. 2010). Colli Albani is considered quiescent at present (Funiciello et al. 2003; De Benedetti et al. 2008; Giordano et al. 2010; Mazza and Capelli 2010); however, persistent gas emissions, ground uplift, anomalous heat flow, active deformation, and periodic seismic swarms reflect possible activity from magmatic bodies at depth (Anzidei et al. 1998; Funiciello et al. 2003; Bianchi et al. 2008; De Benedetti et al. 2008; Giordano et al. 2010; Trasatti et al. 2018; Bisson et al. 2023). Additionally, it has been suggested that the geochemistry of the most recent magmas from Colli Albani may be compatible with a rejuvenation of volcanism (Boari et al. 2009; Giordano et al. 2010), which could lead to violent explosive eruptions linked to the reactivation of long-dormant volcanoes (Bisson et al. 2023). For these reasons, Colli Albani is listed among the ten most active volcanoes in Italy, according to the Italian Civil Protection (Bisson et al. 2023).

For this study, we focus on two eruptive units: the effusive to mildly explosive SLV Formation and the highly explosive VSN Formation. Samples were collected from two main localities on opposite sides of the caldera: Tuscolo and Artemisio (Fig. 1). The SLV Formation represents a phase of effusive to mildly explosive volcanism and is exposed only at the Tuscolo and Artemisio sections, located on opposite sides of the caldera (Fig. 1). The SLV deposits consist of plane-parallel fallout beds (Fig. 2a), non-welded to partially welded scoria and spatter layers, and rare lava flows. Fallout beds are commonly interbedded with reworked volcaniclastic deposits and show frequent signs of post-depositional alteration, often transformed into paleosols. Occasional volcanic bombs and thin pyroclastic density current (PDC) deposits (Fig. 2c) are also observed. Stratigraphically, SLV is divided into two fallout-dominated subunits: a basal fallout (SLVb) and an overlying top fallout (SLVt), separated by a 30 cm paleosol (Fig. 1). The juvenile clasts within SLV are predominantly grey to dark brown scoria clasts from aphyric to sub-aphyric, with small crystals of leucite and clinopyroxene, and typically foidite and phonolitic tephrite in composition (Conticelli et al. 2010). For this study, we selected minimally altered scoriae from both SLVb and SLVt, as these best preserve the original magmatic composition and eruptive textures.

The VSN Formation is stratigraphically separated from the SLV deposits at Tuscolo by a 1.4 m paleosol (Fig. 1) and begins with a basal unit (VSN0) composed of a scoria lapilli fallout and, locally, a surge deposit a few centimeters thick with low-angle cross-laminations and fine sorting. VSN0 extends more than 20 km from the caldera and reaches up to 1.3 m in thickness in proximal areas (Giordano et al. 2010). It is overlain by the Tufo Lionato Ignimbrite (VSN1), a massive, structureless, reverse-graded ignimbrite with a characteristic yellow to orange-red color due to zeolite alteration. This unit is ash-rich and matrix-supported, with abundant aphyric scoriae and minor spatter and lava lithics. The upper ignimbrite, Pozzolanelle (VSN2), is dark grey to dark red, unstructured, and only weakly zeolitized. It is also matrix-supported and consists of coarse ash, crystal fragments, and scoria clasts ranging from aphyric to highly porphyritic. In some sections, a co-ignimbrite breccia (VSN2b) is observed (Fig. 2b), containing juvenile blocks up to 2 m in size along with lava lithics, carbonate fragments, and leucite–clinopyroxene cumulates. The VSN juvenile scoriae range in composition from tephritic to tephritic-phonolitic (Conticelli et al. 2010), and the crystal cargo is dominated by clinopyroxene and leucite, both in the matrix and as phenocrysts. As in SLV, pervasive zeolitization affects parts of VSN1, particularly in Artemisio, which limits the potential for bulk rock geochemistry. For this study, we selected only fresh juvenile pyroclasts from the basal fallout (VSN0) and ignimbrite deposits (VSN1 and VSN2), prioritizing minimally altered scoriae for petrographic and geochemical analysis. Detailed field observations are summarized in supplementary material S1.

## Methods

### Sampling

A total of 20 samples were collected from the Tuscolo and Artemisio sections, targeting stratigraphic variability and minimally altered material. Samples were collected from the base, middle, and top of each stratigraphic unit to capture vertical variability. Although occasional lavas and volcanic bombs were observed in the SLV deposits, for this study, we focused exclusively on juvenile scoriae from fallout and scoria beds, as they were the most representative samples from each subunit and because they preserve better primary magmatic features. In the VSN Formation, we selected scoriae from the basal fallout (VSN0) and juvenile clasts from the ignimbrite deposits (VSN1, VSN2), prioritizing fresh, unaltered pyroclasts. All samples were collected in labeled bags and logged with stratigraphic and lithologic context to ensure traceability.

### Petrography

Thirty-seven polished thin sections were prepared and analyzed under a petrographic microscope (Leica Leitz DMRXP with an integrated Olympus DP26 camera). From selected samples, three thin sections per sample were prepared to control the variability within the same event. We collected information on the textures, size, and shape of the crystals and vesicles. Modal proportions of minerals were determined using a point counting method with 300 points per thin section, using JMicroVision (version 1.3.4; Roduit 2020) to create a statistical record of the relative proportion of the crystals (supplementary material S2). This point count strategy follows the recommendations of Van der Plas and Tobi (1965) and allows an estimate of 2$\sigma$ error ranging from ±1% to ±6%, depending on mineral abundance.

### Mineral separation

Samples were crushed and sieved, and the size fraction of 300 $\mu$m – 1 mm was kept for sorting. A magnetic barrier laboratory separator, Frantz-model LB-1, was used to obtain concentrates based on the magnetic properties of the minerals. Pyroxenes and some amphiboles were separated in the magnetic section, and the non-magnetic part included mainly leucites. The separates were then hand-sorted in an ethanol bath. A total of 153 clinopyroxene crystals were hand-picked and separated to perform further analyses.

### Electron probe micro-analyzer (EPMA) measurements

A JEOL JXA 8200 superprobe hosted at the University of Geneva was used to measure major elements (Si, Ca, Na, Mn, Fe, Al, Ni, Mg, Ti, Cr) in clinopyroxenes. Core-to-rim profiles were taken in the Cpx to record possible chemical variations during the growth of the crystals. The count times and standards used for the measurements are listed in supplementary material S3. Clinopyroxenes were analyzed using a focused beam at 20 nA and 15 kV. Analytical spots with oxide totals below 98 wt.% or above 102 wt.% were rejected. We followed standard operating procedures, from which the errors are typically SiO$_2$ ± 0.07 wt.%, Na$_2$O ± 0.01 wt.%, FeO ± 0.03 wt.%, Al$_2$O$_3$ ± 0.02 wt.%, CaO ± 0.03 wt.%, MgO ± 0.05 wt.%, TiO$_2$ ± 0.01 wt.%, and < 0 wt.% for MnO, NiO, Cr$_2$O$_3$ (Jorgenson et al. 2024). In total, we performed 5113 EPMA determinations, which are reported in the supplementary material S4.

### Laser ablation-inductively coupled plasma mass spectrometry (LA-ICP-MS) measurements

Trace element compositions on clinopyroxenes were measured using single spots and compositional maps. The single spots were taken using an Analyte G2 laser ablation system, interfaced with an iCAP-Q, quadrupole-based ICP-MS (Petrelli et al. 2016) hosted at the University of Perugia. The laser was operated using the software Chromium (Teledyne Photo Machines, Bozeman, MT USA) and the ICP-MS using the Qtegra software (Thermo Fisher Scientific). The spot diameters were set up at 15, 20, 25, and 30 microns, with shot counts of 300 and 350 depending on the size of each crystal. The repetition rate was set to 10 Hz. The He-carrier gas flow was set at 0.6 and 0.3 l min$^{-1}$ in the cell and cup, respectively. Data reduction was performed using the software Iolite (Paton et al. 2011). NIST-SRM610, Ca, and USGS-BCR2G were used as calibrator, internal standard, and quality control, respectively. Under this configuration, precision and accuracy are better than 10% for all the analyzed elements (Petrelli et al. 2016). In total, we performed 264 LA-ICP-MS analyses matching relevant EPMA determinations in order to have a complete major plus traces suite of single-spot data (supplementary material S5).

Compositional maps were acquired using an LA-ICP-MS hosted in the Department of Analytical Chemistry at the National Institute of Chemistry, Ljubljana, Slovenia (Van Elteren et al. 2019). The laser ablation system is an Analyte G2 with 193 nm ArF from Teledyne Photon Machines (Bozeman, MT USA) with a two-volume ablation cell (HelEx II). The He carrier gas flow rate was set up at 0.45 L min$^{-1}$ for the cup and the cell. The LA was interfaced with a quadrupole Agilent 7900 (Agilent Technologies) ICP-MS instrument. Maps were acquired using an aerosol rapid introduction system (ARIS) with a smoothing chamber addition and a non-cyclonic adapter from Glass Expansion. NIST-SRM612, Ca, and USGS-BCR2G were used as calibrator, internal standard, and quality control, respectively. Data reduction and image reconstruction were performed with the software HDIP (Teledyne Photon Machines, Bozeman, MT USA). Typical conditions used during mapping were spot sizes of 5 µm and 10 µm, scan speeds of 50 µm/s and 100 µm/s, and repetition rates of 100 Hz. Detailed analytical conditions used for each map are reported in supplementary material S6. The raw data is reported in supplementary material S7 and can be plotted using the Jupyter Notebook shared as supplementary material S8.

### Cluster analysis

Clustering is an unsupervised machine learning technique used to categorize multivariate data into distinct groups (“clusters” - Templ et al. 2008; Musu et al. 2023; Petrelli 2023, 2024). We applied k-means clustering to trace element compositional maps using the “stats” package in R (R Core Team 2021). Elements used for clustering were Cr, Ni, Sr, Ti, V, Zr, and Sc. The raw data (ppm values per pixel) were merged with single-spot analyses (supplementary material S9, to plot use supplementary material S8). We use elemental ratios (concentrations divided by Ni, followed by logarithmic transformation) to avoid analytical biases (Rollinson 1994; Petrelli 2023). Then, a log-transformation has been applied to reduce data skewness, maintaining the interpretability of the dataset. Zeros (i.e., values below the detection limit) were replaced with a random number between zero and the element’s minimum recorded value. The data were then normalized using a standard scaler to prevent low-abundance elements with high variance from dominating the clustering results (Templ et al. 2008). The “optimal” number of clusters was determined by iteratively testing different scenarios, from six to ten, and visually comparing the resulting cluster maps with BSE images. We first observed that changing the number of clusters within this range did not significantly alter the overall results. We selected eight clusters because this configuration best matched the crystal zoning patterns observed in the maps, without over-segmenting chemically similar regions. Fewer than eight clusters tended to oversimplify the zoning, while more than eight began introducing redundant groups that did not correspond to distinct textures (Templ et al. 2008; Musu et al. 2023).

Major elements were initially excluded from the cluster analysis primarily due to differences in data structure and analytical constraints. Majors, trace element single spot analyses, and trace element maps have been acquired using different approaches, resulting in a non-homogeneous data structure. In particular, major element data analyses have correspondence with trace element single spots, but not with maps. Technically, it is feasible to join trace element maps and trace element single spots for clustering using the common elements (i.e., the trace elements analyzed during mapping and single spots). To associate major elements with a specific cluster, we decided to use data fusion, consisting of matching the trace element single spots with major element data.

**Fig. 3:**
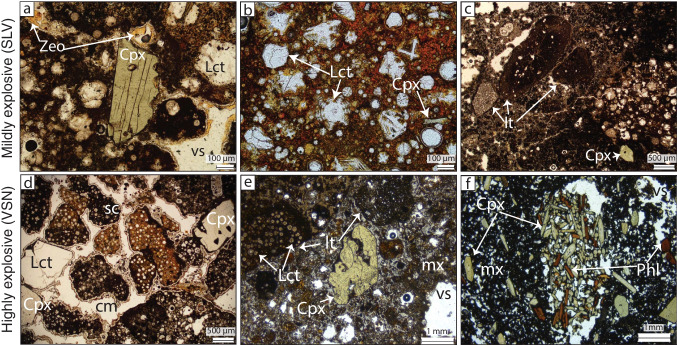
*Optical microscope images*. Microphotograph images of polished thin sections in plane parallel Nicols and transmitted light from the mildly explosive products—SLV (upper panel) and the highly explosive products—VSN (bottom panel). Note the difference in size between the Cpx from the mildly explosive unit—SLV and the highly explosive unit—VSN. Cpx, clinopyroxene; Lct, leucite; Phl, phlogopite; sc, scoria; lt, lithics; Zeo, zeolites; vs, vesicle; cm, cement; mx, matrix

**Fig. 4:**
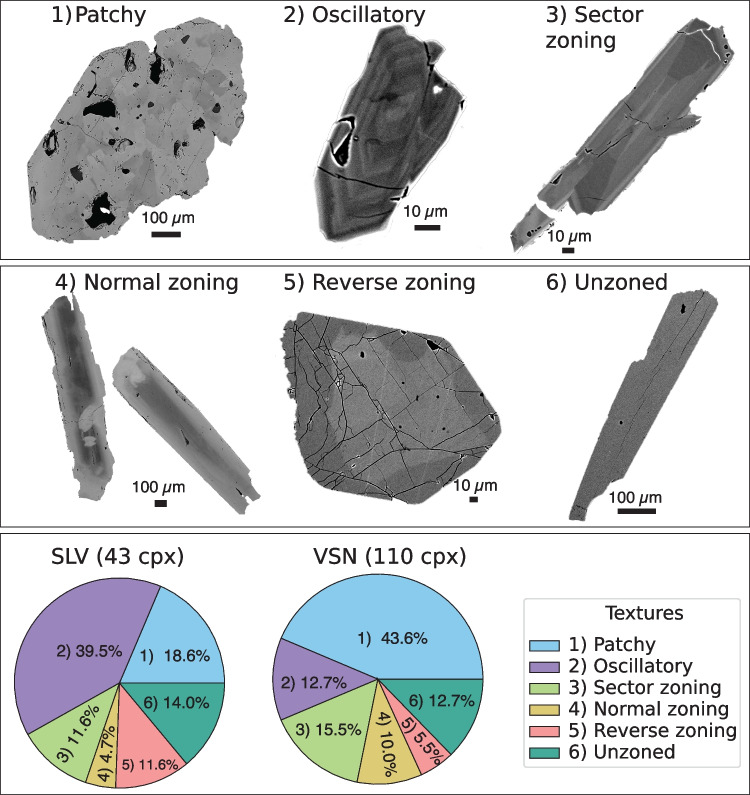
*Clinopyroxene textures and textural distributions*. The first two panels show BSE images from selected Cpx, displaying the different zoning patterns found among the investigated units. The bottom panel shows two pie charts with the distribution of the different zoning found in SLV and VSN Cpx crystals. SLV, Fontana Centogocce Formation; VSN, Villa Senni Formation

### Machine learning (ML) thermobarometry

Pressure and temperature estimates were calculated using the ML-based Cpx thermometer and barometer of Ágreda-López et al. (2024). This model was selected because its training dataset also includes mafic to ultrapotassic compositions, making it well suited for the Colli Albani compositions. This approach is also consistent with recent recommendations for thermobarometry in alkaline systems, where ML-based methods have been shown to yield realistic results across a wide range of undercooling conditions and for phenocrysts with complex zoning and resorbed cores (MacDonald et al. 2023). The thermobarometer estimates pressure (*P*) and temperature (*T*) based on the clinopyroxene chemistry. In detail, an extremely randomized trees algorithm (Geurts et al. 2006) was trained by using SiO$_2$, Al$_2$O$_3$, FeO, MgO, CaO, TiO$_2$, MnO, Na$_2$O, Cr$_2$O$_3$ measurements on experimentally crystallized Cpx (n=1782). Additionally, a series of pre-processing filters (including data scaling, data augmentation, feature engineering, and treatment of data imbalance and equilibrium filter) were applied to the initial dataset to ensure quality data. Then, the analytical errors are propagated through the model using a Monte Carlo simulation. Finally, a bias correction function is applied to mitigate the effect of the “regression to the mean” phenomenon (Zhang and Lu 2012), which is an intrinsic bias observed in tree-based algorithms. The final prediction for each input instance is reported as the median of the distribution of 1000 predictions generated by the Monte Carlo simulation, and the error bars represent the range between the 16th and 84th percentiles of that distribution.

## Results

### Petrography and textures

The majority of SLV samples are vitric tuffs composed of leucite ($\sim$80%) and clinopyroxene ($\sim$20%). Amphiboles and oxides occur as accessory phases (< 0.5%). The matrix is dark brown to orange, consisting predominantly of altered glass (clays and zeolites) with minor microliths of Lct and Cpx (Fig. 3b). Leucite occurs primarily as skeletal crystals (Fig. 3b), while Cpx appears as either small tabular euhedral crystals (in average 100 $\mu$m along the longest axis and 20 $\mu$m along the shortest; Fig. 3b) or larger (up to 800 µm along the longest axis), subhedral to anhedral crystals exhibiting resorption surfaces and fractures. In contrast, PDC deposits have an altered glass matrix (mainly clays) with Lct microliths. The main phenocrysts are Lct and larger subhedral green Cpx phenocrysts (1 mm by 0.5 mm; Fig. 3a). These deposits also contain volcanic and, less commonly, plutonic lithics not observed in the fallouts (Fig. 3c).

In VSN, the basal fallout (VSN0) is stratified; the bottom is a clast-supported reworked deposit constituted by rounded lapilli (2 mm average diameter) with microcrystalline cement filling voids (Fig. 3d). Clasts consist of an altered volcanic glass matrix with embedded Lct phenocrysts (average diameter of 50 $\mu$m) and occasional larger Cpx ($\sim$200 $\mu$m in diameter). Towards the middle of the fallout, there are larger, subhedral and reabsorbed Cpx (average 400 $\mu$m diameter) and Lct phenocrysts (500 $\mu$m diameter). Towards the top, volcanic clasts increase in size (3 mm on average) and become more vesicular compared to those in the lower sections. The matrix also includes Cpx and Lct phenocrysts, averaging 300 $\mu$m in diameter.

VSN1 is a pyroclastic flow deposit composed of 80% scoria, 15% of lithics, and < 5% of sparse phenocrysts. The main phases are leucite ($\sim$70%) and clinopyroxene ($\sim$30%). Amphiboles and oxides occur as accessory phases (< 0.5%). The matrix is brown and altered to clays and zeolites. The matrix also contains some fragmented Lct and Cpx crystals. Clinopyroxenes crystals often exhibit resorption surfaces (Fig. 3e).

VSN2 is the upper ignimbrite subunit and consists of scoria (85%), lithics (10%), and loose crystals (5%). The main modal composition is clinopyroxene (50–60 %), phlogopite (20–30%), and leucite ($\sim$20–30%). Smaller Lct and Cpx crystals are euhedral, whereas many of the larger phenocrysts are fragmented. Crystal sizes typically range within the millimeter scale, though some Lct phenocrysts can reach centimeter sizes. Phlogopite (Phl) occurs as dark brown to reddish crystals exhibiting subhedral to euhedral habits. Aggregates of Cpx and Phl are frequently observed (Fig. 3f). In general, there is not a systematic increase in total phenocryst content from the effusive–mildly explosive SLV units to the explosive VSN units. However, there is a clear shift in modal mineralogy, with Cpx abundance increasing and Lct decreasing from SLV to VSN (supplementary material S2).

**Fig. 5:**
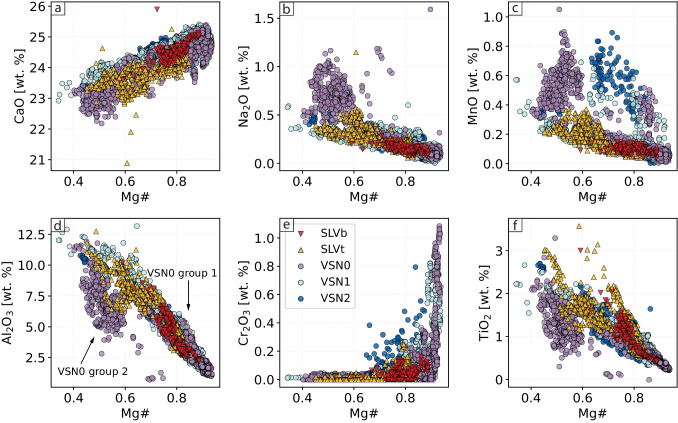
*Clinopyroxene major elements composition*. Binary diagrams displaying the major element variations for clinopyroxenes from SLV (SLVb, SLVt) and VSN (VSN0, VSN1, VSN2)

In all investigated samples, the melt phase is extensively altered to zeolites and clay minerals. Therefore, the melt phase cannot provide reliable information on the architecture and evolution of the magma feeding system. In contrast, the crystal cargo does not show extensive evidence of alteration, except for analcimization observed in some Lct crystals. Thus, crystal phases are better suited for tracing the evolution of the volcanic plumbing system. We focus on the Cpx phase because (i) it is stable across a wide range of pressures, temperatures, and melt compositions, making it useful for reconstructing the crystallization history and evolution of magmatic systems; (ii) its variable zoning patterns record different magmatic processes, allowing changes in magma composition, temperature, and pressure over time to be deciphered; and (iii) well-established modeling tools, such as geo-thermobarometers, are available, enabling robust interpretation of clinopyroxene-bearing systems (Streck 2008; Putirka 2008; Armienti et al. 2013; Mollo et al. 2015; Ubide and Kamber 2018; Mollo et al. 2017; Ubide et al. 2019b, a; Masotta et al. 2020; Neave et al. 2019; Arzilli et al. 2022; MacDonald et al. 2023, 2024a, b; Jorgenson et al. 2024; Ágreda-López et al. 2024).

Using backscattered electron (BSE) imaging, we identified six zoning patterns in the Cpx phenocrysts: (1) patchy, (2) oscillatory, (3) sector zoning, (4) normal, (5) reverse, and (6) unzoned. Figure 4 highlights the distribution of textures across different crystals and units. Figure 4 shows that although all textural types occur in both SLV and VSN, oscillatory-zoned Cpx crystals are most abundant in SLV (39.5%, 17 crystals), followed by patchy (18.6%, 8 crystals), unzoned (14.0%, 6 crystals), sector-zoned (11.6%, 5 crystals), reverse-zoned (11.6%, 5 crystals), and finally, normal-zoned (4.7%,  2 crystals). In contrast, patchy zoning is the predominant texture in the VSN unit (43.6%,  48 crystals), followed by sector zoning (15.5%, 17 crystals), oscillatory zoning (12.7%,  14 crystals), unzoned (12.7%, 14 crystals), normal zoning (10.0%, 11 crystals), and finally reverse zoning (5.5%, 6 crystals).

**Fig. 6:**
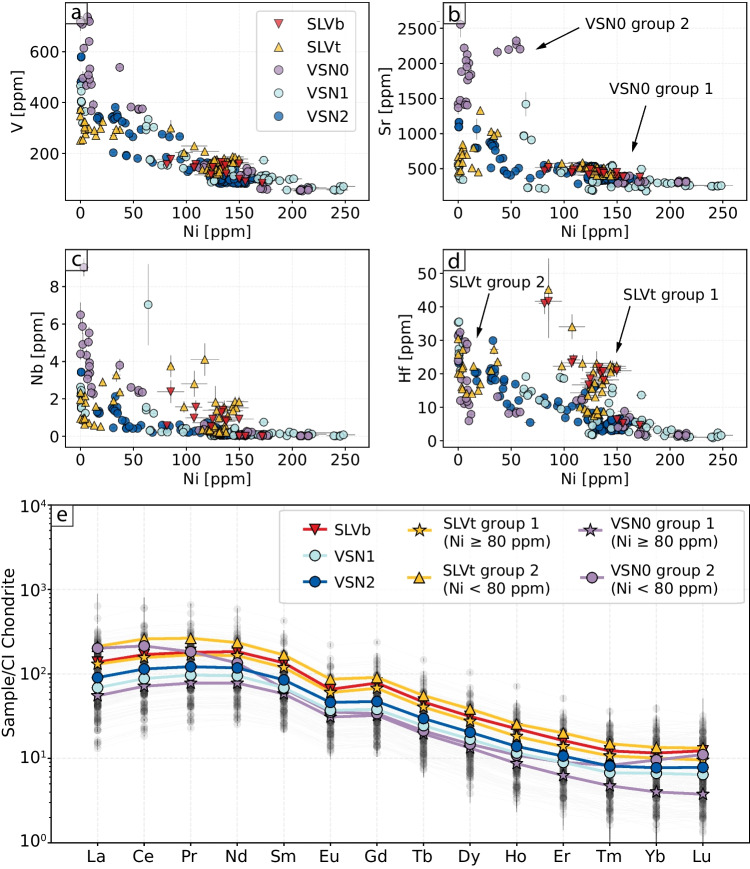
*Clinopyroxene trace elements composition*. SLV and VSN clinopyroxenes trace element composition represented as **a**–**d** binary plots displaying V, Sr, Nb, and Hf vs Ni and **e** spider plots of Cpx showing the rare earth elements (REE) distribution normalized using the CI chondrite values from McDonough and Sun (1995)

**Fig. 7:**
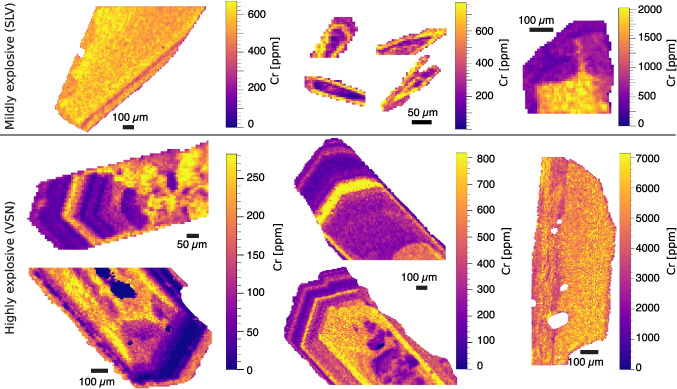
*Clinopyroxene chromium maps*. Chromium distribution for selected crystals from SLV (top) and VSN (bottom)

An important textural observation is the variation in crystal habit and size. In SLV, Cpx crystals are generally smaller (on the $\mu$m scale) than those in VSN (on the mm scale). Two main groups of Cpx can be distinguished in SLV: (1) smaller, euhedral crystals ($\mu$m scale) and (2) larger, subhedral crystals (up to mm scale) exhibiting distinct resorption surfaces. In contrast, Cpx crystals in VSN are consistently larger (mm-scale) and frequently fragmented. They predominantly exhibit patchy zoning and occasionally show resorption surfaces, suggesting a more complex crystallization history compared to crystals in SLV.

### Micro-analytical determinations and thermobarometry

#### Major elements

Figure 5 reports major element chemical variations as a function of Mg# [Mg/(Mg + Fe), calculated here using cations] for SLV and VSN Cpx crystals. SLV and VSN are subdivided into their main stratigraphic subunits (i.e., SLVb, SLVt, VSN0, VSN1, VSN2). The majority of the analyzed Cpx crystals are high-Ca diopsides. The dataset shows a clear positive correlation between Mg# and CaO and a negative correlation between Mg#, Na$_2$O, MnO, Al$_2$O$_3$, and TiO$_2$. SLV plots similarly to VSN1 and VSN2 for most elements with the exception of MnO and Cr$_2$O$_3$ (Fig. 5c, e). Cpx crystals from SLVb exhibit Mg# values ranging from $\sim$0.6 to 0.85, whereas SLVt crystals display a broader Mg# range ($\sim$0.45$-$0.8). VSN0 can be further divided into two distinct groups. The first group (group 1), with Mg# between $\sim$0.6 and $\sim$0.9, follows the same trends observed in most SLV, VSN1, and VSN2 crystals. The second group (group 2), however, exhibits a markedly different composition, with Mg# between 0.4 and 0.6, higher Na$_2$O and MnO, and lower Al$_2$O$_3$ and TiO$_2$ compared to SLV and the majority of VSN1 and VSN2 crystals (Fig. 5). Additionally, in VSN Cpx, Cr$_2$O$_3$ increases with Mg#, particularly for values above 0.80. Such high Cr$_2$O$_3$ and Mg# values are never observed in Cpx from SLV (Fig. 5).

**Fig. 8:**
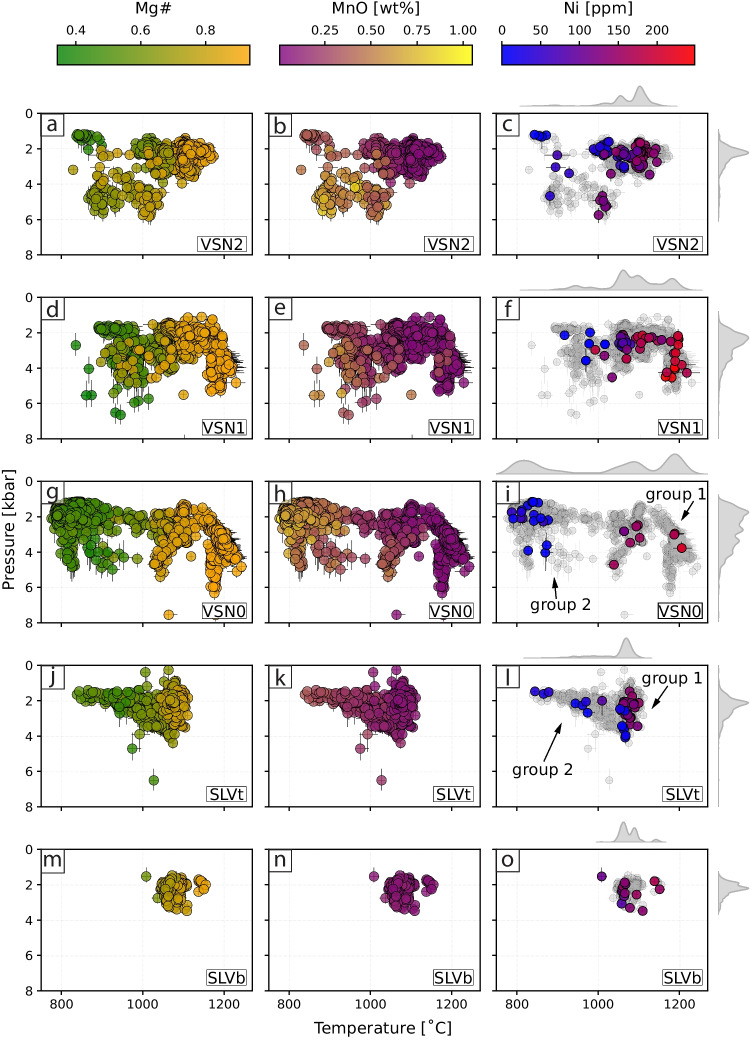
*Clinopyroxene thermobarometry*. Predicted pressure vs. predicted temperature for the different SLV and VSN subunits, arranged from bottom to top according to their stratigraphic distribution. The left panel presents *P-T* estimates color-coded by Mg#. The middle panel shows *P-T* estimates color-coded by MnO [wt.%]. The right panel displays all *P-T* estimates in grey, with Ni concentrations [ppm] from LA-ICP-MS spot analyses overlaid

#### Trace elements

In addition to major elements, trace element analyses provide further insight into crystal compositional variability and zoning. Figure 6 displays the trace element compositions of Cpx from the investigated samples, shown as binary diagrams of V, Sr, Nb, and Hf vs. Ni. The data reveal two key trends: (i) VSN1 and VSN2 show a general continuous trend from higher to lower values for all the reported elements relative to Ni; (ii) SLVt and VSN0 form two distinct compositional groups.

Within SLVt, the first group (group 1) exhibits higher Ni concentrations ($\sim$80–170 ppm), similar to SLVb, whereas the second group (group 2) shows lower values ($\sim$0–50 ppm). In addition, the second group is enriched in V ($\sim$200–400 ppm) and Sr (up to $\sim$1400 ppm) compared to the first group, which has V and Sr concentrations of $\sim$100–200 ppm and $\sim$500 ppm, respectively. Furthermore, the first group (group 1 - Ni: $\sim$80–170 ppm) exhibits higher Nb and Hf concentrations than the second group and all other units at comparable Ni contents (Fig. 6a, b). Similarly, VSN0 can also be subdivided into two groups based on Ni content. The first group (group 1), with Ni values ranging from $\sim$125 to 225 ppm, follows the same trend as VSN1 and VSN2 within this range. The second group (group 2), characterized by Ni values below 50 ppm, is enriched in V, Sr, and Nb relative to all other units (i.e., SLV, VSN1, and VSN2; Fig. 6a–c). The different compositional groups identified in SLVt and VSN0 Cpx crystals reflect differences between crystal populations rather than different zones within crystals (i.e., core, mantle, rim).

Figure 6e reports the chondrite-normalized rare earth elements (REE) patterns for the investigated Cpx crystals (normalization values from McDonough and Sun 1995). Overall, SLV Cpx crystals are, on average, more enriched than VSN samples, although they show a pattern parallel to that of VSN1 and VSN2 (Fig. 6e). Additionally, in SLVt, group 1 Cpx is relatively less enriched in REEs than group 2.

A more detailed analysis of the VSN subunits reveals that VSN0 exhibits two distinct REE patterns, reflecting the two previously described Cpx groups. VSN0 group 1 exhibits lower REE concentrations compared to group 2, displaying a generally parallel trend to all other units, but with a slight depletion in heavy rare earth elements (HREE). In contrast, VSN0 group 2 displays a markedly different REE pattern compared to all other subunits. Specifically, these Cpx are enriched in light rare earth elements (LREE; La, Ce, and Pr) and show high Tm/Er, Yb/Er, and Lu/Er ratios relative to both VSN0 group 1 and all other units. The heavy REE (i.e., Er, Tm, Yb, Lu) are enriched compared to middle REE, which is characteristic of alkaline evolved systems (Baudouin et al. 2020; Simpson et al. 2025).

To better understand the textural and chemical variations within and between different crystals, we acquired quantitative elemental maps for 80 Cpx crystals using the LA-ICP-MS. Figure 7 shows Cr distribution maps for selected Cpx crystals from SLV and VSN. The Cr zoning is extremely useful to understand magma history and mafic recharge/rejuvenation as an eruption trigger, as shown in other mafic alkaline volcanic systems (e.g., Ubide and Kamber 2018; MacDonald et al. 2024b). Overall, SLV Cpx crystals exhibit a relatively simpler Cr distribution (mostly oscillatory zoning) compared to those from VSN. The maximum Cr content in SLV crystals reaches $\sim$2000 ppm, whereas VSN crystals attain values up to 7000 ppm Cr, consistent with EPMA analyses (Fig. 5). Importantly, LA-ICP-MS maps reveal a key feature: high Cr concentrations in VSN Cpx are either restricted to crystal rims or homogeneously distributed throughout unzoned crystals. Additionally, three main crystal groups can be identified in VSN based on Cr content: (i) low-Cr crystals (up to $\sim$300 ppm), typically exhibiting complex zoning patterns; (ii) moderate-Cr crystals (up to $\sim$800 ppm), also displaying intricate zoning but with slightly higher Cr concentrations; (iii) high-Cr crystals (up to $\sim$7000 ppm), characterized by a relatively homogeneous Cr distribution throughout the crystal.

#### Thermobarometry

To complement the chemical and textural analyses, we applied Cpx thermobarometry to estimate crystallization pressures and temperatures across the studied subunits. Figure 8 reports *P-T* estimates for 5113 Cpx analyses from the different SLV and VSN subunits, arranged from bottom to top according to their stratigraphic distribution. The left panel shows *P-T* estimates color-coded by Mg#, while the second panel is color-coded by MnO [wt.%] from EPMA measurements. The third panel displays all *P-T* estimates in grey, with Ni [ppm] from selected LA-ICP-MS spot analyses overlaid. Clinopyroxenes from SLV primarily record pressures between $\sim$1 and 4 kbar and temperatures ranging from $\sim$850 to 1150 $^{\circ }$C. Specifically, Cpx from the basal fallout (SLVb) consistently record high temperatures ($\sim$1100 $^{\circ }$C), whereas SLVt crystals show a broader temperature range ($\sim$850–1150 $^{\circ }$C). Within SLVt, group 1 (Ni $\ge$80 ppm) exhibits higher temperatures ($\sim$1100 $^{\circ }$C), while group 2 (Ni <80 ppm) records lower temperatures ($\sim$850–1000 $^{\circ }$C). In contrast, VSN crystals display a wider range of pressures and temperatures, spanning from $\sim$800 to 1200 $^{\circ }$C and from $\sim$2 to 8 kbar. A few crystals record high pressures ($\sim$4–8 kbar) and temperatures ($\sim$1150–1200 $^{\circ }$C). Within VSN0, group 1 (low MnO, Mg# $\sim$0.8, Ni $\ge$80 ppm) exhibits the highest temperatures ($\sim$1150–1200 $^{\circ }$C) and pressures (up to $\sim$7 kbar), whereas group 2 (high MnO, Mg# $\sim$0.5, Ni <80 ppm) records the lowest temperatures.

## Discussion

### Blending different information

To integrate the results from single-spot analyses with textural information derived from trace element mapping, we performed a combined cluster analysis incorporating both single-spot trace element data (264 observations) and trace element maps (80 crystals). Since each LA-ICP-MS spot was placed on a previously analyzed EPMA spot, we were able to directly correlate trace element concentrations with major element compositions. Through the cluster analysis, each trace-element spot was assigned to a specific cluster, which we then directly linked to its corresponding major-element composition and *P–T* estimates. This process resulted in a combined dataset in which each cluster integrates specific major-element data, trace-element data, and *P–T* estimates.

**Fig. 9:**
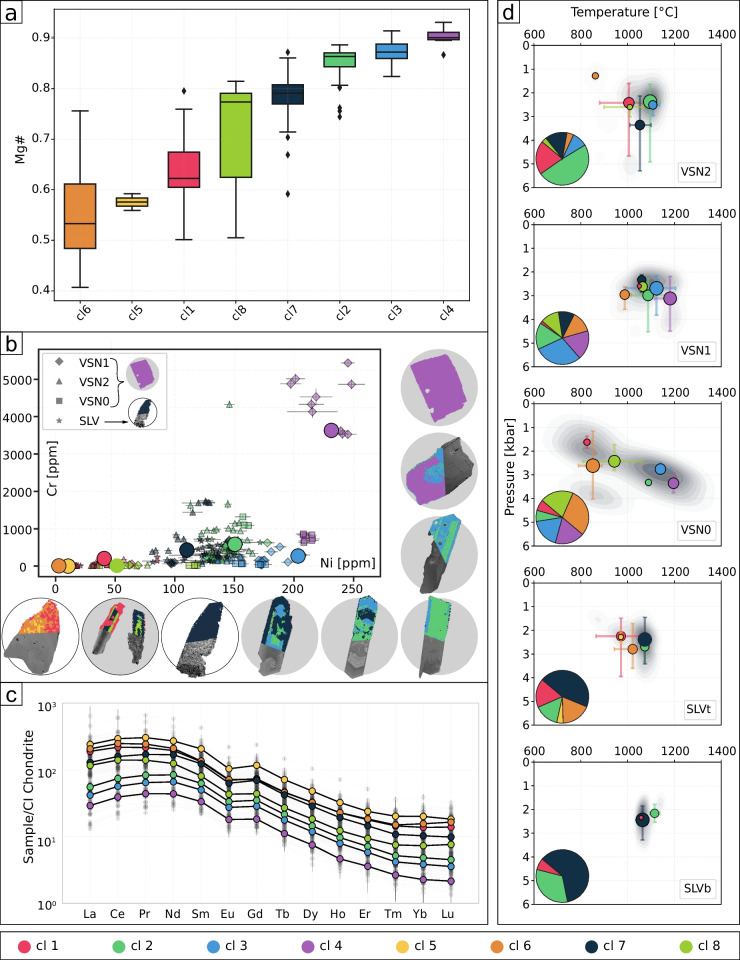
*Blended data using cluster groups*. **a** Boxplots showing the distribution of Mg# for each cluster. **b** Binary plot of Cr vs Ni and selected crystals displaying the distribution of each cluster within and between crystals. Circles represent the average composition of each cluster. **c** Spider plot showing the REE concentration for each cluster. **d** *P-T* estimates for the SLVb, SLVt, VSN0, VSN1, and VSN2 subunits, color-coded by cluster. Circles indicate the average *P-T* values for each cluster, with circle size proportional to the cluster’s abundance in each subunit. Bars represent the *P-T* distribution for each cluster within each subunit. To mitigate the effect of outliers, the bars encompass 98% of the data, including values between the 1$^{st}$ and 99$^{th}$ percentiles for each subunit

Figure 9 presents the results of the cluster analysis, with panel (a) showing boxplots of Mg# distribution for each cluster. Figure 9b illustrates the Cr and Ni contents of each cluster, along with the distribution of textures within and between crystals. Figure 9c presents the average REE pattern for each cluster, while Fig. 9d shows the *P-T* estimates for each subunit (SLVb, SLVt, VSN0, VSN1, and VSN2) along with pie charts illustrating the relative abundance of clusters within each subunit. The distribution of major elements for the 264 observations, color-coded by cluster, is presented in supplementary material S10.

Clusters 3 and 4 (cl3, blue; cl4, purple) represent the most primitive compositions, characterized by higher Ni, Cr and lower REE concentrations (Fig. 9). They also exhibit the highest Mg# and temperatures ($\sim$1200 $^{\circ }$C), with pressures ranging from $\sim$2 to 4.5 kbar. These clusters occur in highly explosive products (i.e., VSN subunits), and we propose them as proxies for Cpx crystallizing from the hottest, least evolved magma. Cluster 2 (cl2, dark green) represents crystals derived from magma compositions slightly more evolved compared to cl3 and cl4 (Fig. 9a and b). Clusters 2 and 3 commonly occur together within individual crystals, defining oscillatory and/or sector zoning textures (Supplementary material S11), and are predominantly observed in highly explosive eruptive products (VSN subunits; Table 1, Fig. 9a, c). Cluster 7 (cl7, black) is the dominant cluster in effusive to mildly explosive products (SLV) and occurs only rarely in highly explosive products (VSN). In SLV, crystals associated with cl7 yield temperature estimates ranging from $\sim$1000 to 1100 $^{\circ }$C and pressures between $\sim$1.5 and 3.5 kbar. However, in the VSN products, pressure estimates reach values as high as 5.5 kbar. We interpret this cluster as representing crystals derived from magma compositions slightly more evolved than those of cl2, cl3, and cl4 within a trans-crustal ($\sim$1.5$-$5.5 kbar) volcanic plumbing system. This system may consist of separate magma batches or a vertically extended crystal mush, with the SLV products recording only the shallower portion.

**Table 1:** Distribution and petrological meaning of the identified clusters in the magmatic plumbing system of the Colli Albani volcano

Cluster	Representative map	Textural distribution	Petrological meaning	Stratigraphic distribution
cl1		Appears mainly in the rims of normal zoned crystals	Crystallization from an evolved and relatively colder magma than the majority of compositions of the system (potential cooling and evolution of magmas represented by clusters 7 (cores) and 8 (mantles) in normally zoned crystals). It is mainly found in VSN2	
cl2		Appears in cores, in oscillatory crystals or in the hourglass of SLV sector zoned crystals or in the prism of VSN Sector zoned crystals	Crystallization deriving from slightly more evolved magma composition than those of cl3 and cl4 within a trans-crustal volcanic plumbing system. It is mainly found in crystals from VSN2	
cl3		Appears mainly in core/mantles of crystals or in the prism of Sector zoned crystals (VSN)	Crystallization from slightly more evolved magma than cl4 (cooling of the hotter body or mixing between an high fraction of hot material and a smaller fraction of more evolved resident magma).It is mainly found in VSN	
cl4		Appears in unzoned crystals or in the rims of crystals	Crystallization from high-*T* primitive magma. Is the least evolved composition of the system (potential deep injection-trigger of VSN eruptions). It is mainly found in VSN0 and VSN1 crystals	
cl5		Appears mainly in cores of crystals or associated with cl6	Crystallization from the most evolved composition of the system (potential residual magma from SLVt eruption). It is mainly found in VSN1 crystals	
cl6		Appears mainly in unzoned crystals or associated with cl5	Crystallization from the most evolved composition of the system (potential differentiation and evolution of the residual magma from SLVt eruption). It is mainly found in VSN0 and VSN1 crystals	
cl7		Appears in rims, cores or in the prism of SLV sector zoned crystals	Crystallization from slightly more evolved magma composition than those of cl2, cl3, and cl4 within a trans-crustal volcanic plumbing system (main source of the SLV eruptions). It is mainly found in SLV and VSN2 crystals	
cl8		Appears mainly in the mantles of crystals	Crystallization from an evolved and relatively colder magma often associated with cl1 and cl7 (potential cooling and evolution of magmas represented by cl2 and cl7. It is mainly found in VSN1 crystals)	

The remaining clusters (1, 5, 6, and 8) represent Cpx crystals crystallized from the most evolved magma compositions, characterized by the lowest crystallization temperatures and a wide pressure range ($\sim$1.1$-$4.7 kbar; Fig. 9c). We interpret these clusters as Cpx compositions originating from cooler magma pockets derived from magmas related to cl7 and cl2, stored at various crustal depths. Alternatively, these clusters might also represent a single, chemically zoned magmatic reservoir, where different zones within the reservoir record different crystallization conditions. Notably, cl6 includes most crystals belonging to VSN0 group 2, characterized by relative enrichment in HREE (Fig. 9c) and higher MnO [wt.%] (supplementary material S10). The distinct REE pattern characterizing cl6 and VSN0 group 2 (Fig. 6e) could be explained by several processes. One possibility is that the observed variations in Cpx REE patterns reflect differences in melt composition recorded during the differentiation process. Specifically, enrichment in heavy REE (HREE) may indicate fractionation of phases such as amphibole. Alternatively, these clinopyroxenes could record assimilation of host rock material, potentially involving carbonate-bearing components. Carbonate assimilation has been demonstrated to modify REE patterns, particularly by enhancing LREE enrichment and altering overall REE fractionation trends (Knuever et al. 2023). An additional and not mutually exclusive explanation is the incorporation of HREE into the M1 crystallographic site, as a typical feature in alkaline evolved Cpx (Baudouin et al. 2020; Simpson et al. 2025). Given that VSN0 group 2 is also characterized by lower Mg# and higher Na$_2$O concentrations, these geochemical signatures could further support either interaction with carbonate-bearing components (carbonate assimilation has been acknowledged as a petrogenetic contributor in the Roman Province; Gaeta et al. 2009; Mollo et al. 2010) or crystallization from an evolved melt influenced by fractionation processes distinct from those affecting the other VSN subunits (Table 1 and supplementary material S11). A summary of average trace element concentrations and their normalized (z-scored) values for each cluster is provided in supplementary material S12, allowing a quantitative assessment of how each element contributes to each cluster.

**Fig. 10:**
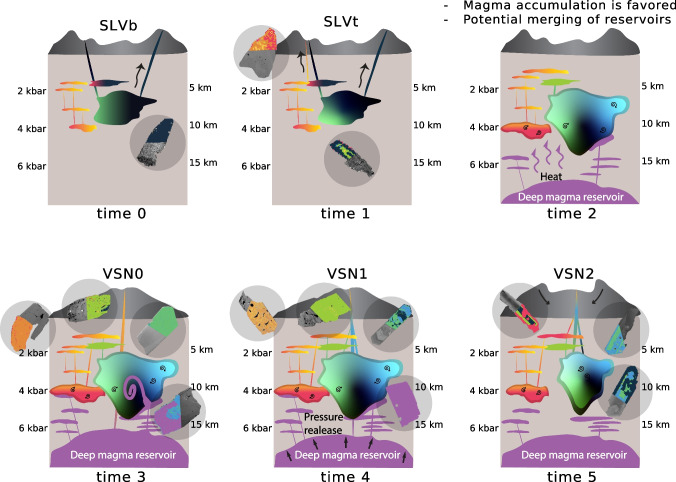
*Schematic model of the magmatic evolution during the last caldera-forming event at Colli Albani*. Conceptual model illustrating the evolution of the magmatic plumbing system at Colli Albani from the SLV (time 0, time 1) to the VSN eruptions (time 3 to time 5)

### Reconstructing the volcanic plumbing system

The first eruption of the investigated sequence is recorded in Cpx from the basal fallout of SLV (SLVb). These crystals are small ($\sim$20x80 $\mu$m) euhedral and predominantly oscillatory zoned, with crystallization temperatures ranging from 1100 to 1200 $^{\circ }$C. Their composition is mainly defined by clusters 7, 2, and 1 (Fig. 9). The presence of idiomorphic, small ($\sim$80 µm) Cpx and abundant skeletal leucite crystals suggests a relatively rapid ascent rate (Shea et al. 2010). Following SLVb deposition, there is a pause in volcanic activity recorded by the presence of a paleosol found in both sampled locations (Fig. 1). Subsequently, the system is reactivated, as recorded by Cpx crystals from the upper SLV deposit (SLVt), in which two distinct compositional groups were identified based on trace element analyses (Fig. 6). Cluster analysis confirms these differences, with group 1 (less evolved) corresponding to clusters 2 and 7, and group 2 (more evolved, i.e., lower Cr, Ni and Mg# values, higher Sr and REE concentrations; Fig. 9a-c) corresponding to clusters 1, 5, and 6.

Group 1 Cpx in SLVt corresponds compositionally to SLVb Cpx, with small crystals ($\sim$20x60 $\mu$m) present alongside larger phenocrysts ($\sim$200x800 $\mu$m) not observed in SLVb. In contrast, group 2 Cpx is only found in SLVt, particularly in the Tuscolo location (Fig. 1). These crystals are larger ($\sim$300x900$\mu$m to mm scale), subhedral, and patchy zoned with lower crystallization temperatures $\sim$980–1000 $^{\circ }$C compared to group 1 $\sim$1100–1200$^{\circ }$C (Fig. 9d). We interpret group 1 and 2 Cpx crystals as crystallized from a heterogeneous magmatic system. In this scenario, Cpx crystals from group 1 could have formed under varying degrees of undercooling or experienced a more complex history, recording a wide range of pressures (SLVt group 1 pressure ranges from $\sim$0 to 4 kbar; Fig. 8), with larger crystals indicating longer residence times within the system. On the other hand, group 2 Cpx crystals originated from a more evolved magma batch (cl1, cl5, cl6) before interacting with a hotter, less evolved magma (cl2, cl7), resulting in the resorption textures observed on their crystal rims.

The combination of petrological and volcanological evidence (such as the presence of volcanic bombs at the Artemisio location and the lack of grain-size distribution gradation towards the Tuscolo location) suggests that the SLV deposits were likely produced by frequent but small eruptions, possibly linked to independent scoria cones or centers rather than a single vent (time 0-time 1; Fig. 10). This interpretation is consistent with existing literature, which describes the SLV as a prolonged phase of mildly explosive and effusive eruptions from peri-caldera centers, followed by violent Strombolian to Plinian eruptions from the central collapsed caldera after the paroxysmal eruption of the Pozzolane Nere ignimbrite (PNR 10 km$^3$ DRE outflow and 5 km$^3$ DRE intracaldera; Giordano et al. 2010).

Following the SLV deposition, a quiescent period is indicated by a 1.4 m thick paleosol found between SLV and VSN0 (Fig. 1, time 2 Fig. 10). While the precise duration is difficult to determine, moderately developed paleosols 1–2 m thick are generally interpreted to represent several thousand to tens of thousands of years of surface stability, depending on climate and parent material (Retallack 2001). In volcanic terrains, for instance, Andisols of 1$-$1.5 m thickness have been shown to develop over approximately $\sim$5–15 ka under temperate to subhumid conditions (Solleiro-Rebolledo et al. 2015). This period of volcanic quiescence was followed by a major shift in the magmatic regime.

The transition to the “high energy” phase of the system, which culminated in the caldera-forming eruption (i.e., VSN), involved the injection of more mafic magmas represented by clusters 3 and 4 (Table 1, time 3; Fig. 10). These clusters are identified in VSN0 and VSN1, recording the arrival of hotter, less evolved magma into the volcanic plumbing system. The new magma interacted with the more evolved magma (represented by all other clusters) already residing within the volcanic plumbing system. This interaction is recorded by reverse zoning, patchy textures, and crystal resorption. Patchy Cpx textures may result from diffusion-limited crystal growth followed by subsequent overgrowth due to magma mixing or reheating (Welsch et al. 2016; Brehm and Lange 2020; Jorgenson et al. 2024), consistent with the introduction of fresh magma into the system. We propose that the injection of this new magma ultimately triggered the VSN eruptions, beginning with the deposition of the basal fallout unit (VSN0) (Vinkler et al. 2012; Jorgenson et al. 2024). VSN0 exhibits the greatest variability in compositions and in *P-T* estimates, recording crystallization temperatures ranging from $\sim$800 $^{\circ }$C to $\sim$1200 $^{\circ }$C. The ascent of mafic magma likely destabilized the shallower magmatic reservoir(s). Additionally, an increase in vesicularity toward the top of VSN0 (Section “Petrography and textures”) indicates enhanced volatile exsolution, preceding the major caldera-forming eruption and subsequent emplacement of the VSN ignimbrites. All of this is in agreement with the lower proportion of melt inclusions with a high volume fraction observed for VSN0, indicative of deep magma and possibly rapid ascent (Jorgenson et al. 2025).

Following the VSN0 eruption, a release of pressure due to the removal of magma from the volcanic plumbing system likely triggered additional injection of material from deep sources, as evidenced by the mafic Cpx compositions found for some crystals in VSN1 (cl4; Fig. 9 and Table 1) and also reported by Vinkler et al. (2012). However, our data indicate that the primary volume of the VSN ignimbrites was sourced from a slightly more evolved magma body, distinct from the fresh mafic magma involved at the onset of the eruption. This is consistent with the variable proportions of melt inclusion types found for VSN1, interpreted as a variation of deposit type or possibly magma deceleration (Jorgenson et al. 2025). This evolved magma accumulated at mid- to shallow-crustal levels (time 4; Fig. 10). The absence of cl4 in the VSN2 Cpx crystals (Table 1) and *P-T* estimates ranging between 2 and 5 kbar (Fig. 9) further support this interpretation.

For the second subunit of the Villa Senni Formation (VSN2), we identified crystal-rich aggregates (Fig. 3f) that could have derived from a crystal-rich mush reservoir. The presence of phlogopite in these aggregates is particularly significant as it typically forms in volatile-rich, potassium-rich magmas, and its stability is favored by the presence of water and other volatiles like fluorine and CO$_2$. Its occurrence here likely reflects increasingly volatile-rich conditions toward the end of the eruption, consistent with a final degassing phase. This is also in agreement with the higher proportion of Cpx relative to Lct observed for VSN2, consistent with Cpx-dominated crystallization under volatile-rich, possibly deeper-sourced magmatic conditions. Additionally, VSN2 is shown to be extremely volatile-rich with volume fractions up to 78%, which is suggested to be indicative of a magmatic reservoir that is rich in exsolved melts prior to eruption (Jorgenson et al. 2025). Ultimately, the sustained magma output likely triggered the caldera collapse (time 5, Fig. 10).

The ML-based *P–T* estimates for SLV ($\sim$1–5 kbar) and VSN (up to $\sim$8 kbar) eruptions are in agreement with previous interpretations of the Colli Albani plumbing system (e.g., Giordano et al. 2010). Geophysical and geochemical monitoring data, such as ground deformation, seismic swarms, and gas emissions, along with geodetic modeling of recent uplift, receiver function analyses, and seismic tomography studies, indicate that present day magma bodies are likely located at $\sim$4–8 km depth (Amato and Chiarabba 1995; Chiarabba et al. 1997; Feuillet et al. 2004; Bianchi et al. 2008; Trasatti et al. 2018). While these observations reflect the current state of the system and cannot directly constrain magma storage conditions during the SLV and VSN eruptions ($\sim$355 ka), they are broadly consistent with our pressure estimates for SLV and VSN clinopyroxenes.

It has been stated that mafic caldera-forming systems can switch from dormancy to eruption relatively rapidly (Annen and Sparks 2002; Sparks et al. 2019; Costa et al. 2020; Petrelli and Zellmer 2020; Jorgenson et al. 2024); however, our data show that in the Colli Albani system, crystal size and zoning complexity increase with time, from SLV (effusive-mildly explosive) to VSN (explosive caldera-forming unit), suggesting that the caldera-forming event was preceded by more protracted magma storage and chemical evolution than the earlier effusive-mildy explosive phase activity. This prolonged storage phase likely allowed for the accumulation and/or evolution of crystal-rich mush zones that were only destabilized shortly before the eruption. Moreover, the ignimbrites also contain larger and more fragmented crystals, which is consistent with both longer residence times and a higher carrying capacity associated with the explosive eruptions. While the final trigger and unrest phase may be rapid, the storage and maturation of magma bodies feeding the caldera-forming event may span longer timescales. These observations highlight the importance of considering both short-term unrest and longer-term magmatic evolution when assessing eruption potential in mafic volcanic systems.

## Conclusions

In this study, we probed the crystal cargo of two key eruptive sequences at Colli Albani volcano: the Fontana Centogocce (SLV) Formation and the Villa Senni (VSN) Formation. By combining fieldwork, petrographic and textural observations, clinopyroxene (Cpx) chemistry, and machine learning-based techniques (thermobarometry and clustering), we constrained the processes and dynamics governing the transition from mildly explosive to highly explosive activity in the studied magmatic sequences.

Our findings indicate that between two major caldera-forming events (PNR and VSN), residual eruptible magma sustained effusive to mildly explosive activity. At that time, magma was likely distributed in the crust between $\sim$1 and 4 kbar in different magmatic reservoirs. These reservoirs fed mildly explosive to effusive eruptions represented by the Fontana Centogocce (SLV) Formation. This idea is supported by field observations and variations in the textures, sizes, and chemistry of the SLV Cpx crystals. The main build-up phase leading to the caldera-forming eruption occurred after the final SLVt eruptions and at the onset of the VSN0 eruption, marked by the injection of primitive, hot magma pulses into the volcanic plumbing system, ultimately triggering the VSN eruptive sequence. The presence of phlogopite in the last ignimbrite (VSN2) points to a high concentration of volatile species in the system during the final period prior to the caldera collapse.

Our results are essential for improving hazard assessments in regions where historical petrological records indicate a potential shift in eruption style from effusive to highly explosive activity in mafic magmas. To better anticipate such shifts, real-time geophysical, geochemical, and petrological monitoring should focus on detecting magma recharge events in shallow storage systems and tracking the evolution of magmatic reservoirs at Moho depths. This would serve to identify the influx of volatile-rich primitive magmas into shallower reservoirs, which might quickly escalate into high-energy explosive eruptions.

This study not only enhances our understanding of mafic caldera dynamics but also provides an initial framework for interpreting the evolution of volcanic plumbing systems that feed catastrophic caldera-forming eruptions in mafic systems. By highlighting the complex interactions between deep and shallow magmatic reservoirs, our findings underscore the importance of targeted monitoring efforts in mafic systems to improve volcanic hazard assessments and better constrain the conditions leading to explosive eruptions.

## Data Availability

All the data (including supplementary material) used in this manuscript are available in the repository Zenodo at the link: https://doi.org/10.5281/zenodo.16777835
